# Increasing mitigation ambition to meet the Paris Agreement’s temperature goal avoids substantial heat-related mortality in U.S. cities

**DOI:** 10.1126/sciadv.aau4373

**Published:** 2019-06-05

**Authors:** Y. T. Eunice Lo, Daniel M. Mitchell, Antonio Gasparrini, Ana M. Vicedo-Cabrera, Kristie L. Ebi, Peter C. Frumhoff, Richard J. Millar, William Roberts, Francesco Sera, Sarah Sparrow, Peter Uhe, Gethin Williams

**Affiliations:** 1 School of Geographical Sciences, University of Bristol, Bristol BS8 1SS, UK.; 2 Cabot Institute for the Environment, University of Bristol, Bristol BS5 9LT, UK.; 3 Department of Public Health, Environments and Society, London School of Hygiene & Tropical Medicine, London WC1E 7HT, UK.; 4 Centre for Statistical Methodology, London School of Hygiene and Tropical Medicine, London, UK.; 5 Center for Health and the Global Environment, University of Washington, Seattle, WA 98105, USA.; 6 Union of Concerned Scientists, Cambridge, MA 02478, USA.; 7 Environmental Change Institute, University of Oxford, Oxford OX1 3QY, UK.; 8 Committee on Climate Change, London SW1W 8NR, UK.; 9 Oxford e-Research Centre, Department of Engineering Science, University of Oxford, Oxford OX1 3QG, UK.

## Abstract

Current greenhouse gas mitigation ambition is consistent with ~3°C global mean warming above preindustrial levels. There is a clear need to strengthen mitigation ambition to stabilize the climate at the Paris Agreement goal of warming of less than 2°C. We specify the differences in city-level heat-related mortality between the 3°C trajectory and warming of 2° and 1.5°C. Focusing on 15 U.S. cities where reliable climate and health data are available, we show that ratcheting up mitigation ambition to achieve the 2°C threshold could avoid between 70 and 1980 annual heat-related deaths per city during extreme events (30-year return period). Achieving the 1.5°C threshold could avoid between 110 and 2720 annual heat-related deaths. Population changes and adaptation investments would alter these numbers. Our results provide compelling evidence for the heat-related health benefits of limiting global warming to 1.5°C in the United States.

## Teaser

Increasing climate commitments to meet the Paris Agreement goal avoids large numbers of heat-related deaths in U.S. cities.

## INTRODUCTION

The nationally determined contributions (NDCs) submitted to the United Nations Framework Convention on Climate Change under the Paris Agreement have been estimated to be consistent with emission pathways reaching a median global mean temperature increase of 2.6° to 3.1°C above preindustrial levels by 2100 (*1*). This estimate excludes conditional NDCs (e.g., financial conditions) and assumes that the nations’ stated 2020–2030 mitigation ambition remains the same through 2100. Although end-of-century warming will be strongly influenced by post-2030 mitigation and the Paris Agreement (*2*) contains a mechanism for nations to progressively “ratchet up” future ambition, a substantial increase in mitigation ambition before 2030 would be required “to keep a global temperature rise this century well below 2°C above pre-industrial levels and to pursue efforts to limit the temperature increase even further to 1.5°C” (*3*). Nations that are parties to the Paris Agreement have a first opportunity to revise their level of mitigation ambition in 2020 (*4*).

Changes in global climate affect human heat-related morbidity and mortality through changes in local ambient temperature and relative humidity. Through the use of statistical models, epidemiological studies (*5*–*7*) have quantified the exposure-response relationship between daily mean temperature and mortality. This relationship varies from location to location, with relationships in developed nations generally “U”- or “J”-shaped, that is, elevated mortality risk at extreme high and low temperatures (*5*, *8*).

Climate change is projected to increase heat-related mortality in the United States (*9*, *10*), Europe (*11*–*13*), the Americas (*13*), East and Southeast Asia (*13*, *14*), Australia (*13*, *15*), and the Middle East and North Africa (*16*). Stabilizing future climate at 1.5°C above preindustrial levels is considerably better than 2°C with regard to heat exposure and heat-related mortality: On average, 73 million fewer Europeans would experience summer temperatures that exceed the 1950–2017 record (*17*); the likelihood of an event with a similar mortality level to that seen in the 2003 European heat wave would be 2.4 times lower in London and 1.6 times lower in Paris (*18*), and people in West Africa would experience dangerous heat stress levels ~2% less often (*19*). Conversely, a global mean temperature rise from 1.5° to 2°C would increase heat-mortality impacts by 0.11 to 2.13% in most countries, with further warming exacerbating these impacts (*20*). The Intergovernmental Panel on Climate Change (IPCC) has medium confidence in detecting the heat-related morbidity and mortality differences between 1.5° and 2°C warming above preindustrial levels (*3*).

Despite a growing understanding of the benefits to human health of keeping warming to 1.5°C compared to 2°C, only one study (*20*) compared temperature-related mortality impacts between the Paris Agreement goal and higher warming levels. No study has yet to delineate the potential benefits of limiting the current trajectory of 3°C warming to the Paris Agreement goal with respect to heat-related mortality or any climate impact, in the framework of extreme event attribution. Addressing this question was outside the purview of the IPCC special report on 1.5°C warming, which was specifically tasked in its approved outline with comparing impacts at 1.5° and 2°C (*3*). Yet, such analysis can inform mitigation discussions in the context of the current international climate ambition. Furthermore, large ensemble experiments are key to assessing the impacts of extreme weather events over policy-relevant return periods (*21*). Although there are challenges in associating near-term NDCs with a particular temperature outcome (*22*), the potential reduction of heat-related mortality associated with achieving the Paris Agreement goal is highly relevant to motivate strengthening of mitigation ambition in the next round of NDC submissions in 2020.

The “Half a degree Additional warming, Prognosis and Projected Impacts” (HAPPI) project (*21*) was established to understand how extreme weather might differ between the recent past and future climates that abide by the Paris Agreement goal. Existing HAPPI experiments include a decade-long experiment spanning 2006–2015 and two time-slice experiments of decades in the future where global mean warming is stabilized at 1.5° and 2°C above preindustrial levels (see Materials and Methods for precise details). In addition to these scenarios, we perform a fourth experiment where global mean temperature stabilizes at around 3°C above preindustrial levels. This allows us to investigate whether and to what extent limiting the 3°C warming consistent with current mitigation ambition to 2° or 1.5°C could avoid heat-related mortality over policy-relevant time scales.

We investigate 15 cities (Atlanta, Boston, Chicago, Dallas, Detroit, Houston, Los Angeles, Miami, New York City, Philadelphia, Phoenix, San Francisco, Seattle, St. Louis, and Washington, DC) covering a range of regions and climates in the United States (*23*). In the following sections, we compare the temperature and heat-related mortality related to 3°C warming with those related to the 2° and 1.5°C Paris Agreement thresholds. We show the projected heat-related mortality avoided by achieving these thresholds, under the assumption of constant population and no changes in vulnerability. We focus on the avoided mortality associated with 1-in-30-year heat events. We suggest that the 30-year return period is more relevant to climate change mitigation and adaptation action than rarer events. Our results provide previously unidentified and notable information for the next round of NDC submissions.

## RESULTS

### Observed and projected changes in extreme high temperature

**Fig. 1:**
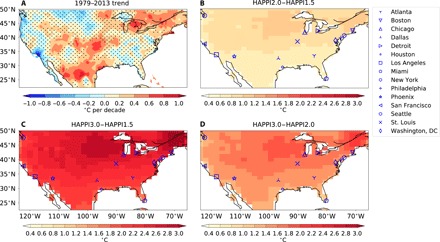
Observed and projected changes in extreme high temperature over the contiguous United States. (**A**) Estimated trends of the annual mean TXx values between 1979 and 2013 from the EWEMBI dataset. (**B**) Difference in the decadal averages of TXx between the 2° and 1.5°C worlds. (**C**) Same as (B) but between the 3° and 1.5°C worlds. (**D**) Same as (C) but between the 3° and 2°C worlds. The differences in (B), (C), and (D) are averages across 30 bias-corrected ensemble members of Hadley Centre Atmospheric Model version 3P (HadAM3P). Stippling indicates regions where neither trends nor differences are significant at the 5% significance level using the two-sigma test and the Kolmogorov-Smirnov test, respectively. The markers indicate the locations of the cities included in this study.

Regional extreme high temperatures that are usually associated with elevated mortality risks have changed over the past decades, often at rates higher than that of increases in global average temperature (*24*, *25*). Figure 1A shows the 1979–2013 trends in annual mean values of monthly maximum of daily maximum temperature (TXx) over the contiguous United States (see Materials and Methods). TXx is a temperature extreme index defined by the Expert Team on Climate Change Detection and Indices (*26*). We estimated the trends from the ﻿EartH2Observe, WFDEI, and ERA-Interim data Merged and Bias-corrected for ISIMIP (EWEMBI) dataset (*27*, *28*) (see Materials and Methods) using the methods described in Sillmann *et al.* (*24*).

Boston, Detroit, Houston, New York City, Phoenix, and St. Louis have experienced a significant warming trend in TXx of up to 2.4°C per decade between 1979 and 2013 (note that stippling on Fig. 1A indicates changes that are not statistically significant at the 5% level). The magnitude, significance, and spatial pattern of these trends are consistent with the trends estimated by Sillmann *et al.* (*24*) for 1971–2010 using another dataset. We bias-corrected the daily maximum temperature simulations in the HAPPI 1.5°, 2°, and 3°C experiments from the atmosphere-only model HadAM3P (*29*, *30*) against the EWEMBI data, to investigate how TXx may change under different levels of warming (see Materials and Methods). For the rest of the study, we bias-corrected the daily mean temperature simulations from the same climate model against the 1987–2000 observations in the National Morbidity, Mortality, and Air Pollution Study (NMMAPS) (*31*) (see Materials and Methods), as this dataset was used to estimate the temperature-mortality relationship in the 15 cities in this study (see the next section). The bias-corrected simulations are referred to as “HAPPI1.5,” “HAPPI2.0,” and “HAPPI3.0” hereafter.

An increase in regional temperature extremes greater than the rise in global and regional mean temperatures has been projected for climate change scenarios over nearly all land regions (*25*, *32*). Figure 1B shows the average differences in decadal mean TXx across 30 ensemble members (see Materials and Methods) between the 1.5° and 2°C Paris Agreement thresholds. A global mean warming of 0.5°C between these thresholds is projected to significantly increase TXx by 0.6° to 1°C in the studied U.S. cities (indicated by various markers in Fig. 1). These values are consistent with those reported for North America in previous studies (*32*, *33*).

Figure 1 (C and D) shows the corresponding TXx differences between a 3°C warmer world, which the current NDCs imply, and the 1.5° and 2°C thresholds. TXx is significantly higher in the 3°C world for all studied U.S. cities, compared to either temperature threshold mentioned in the Paris Agreement. For each 1°C of additional global mean warming resulting from current mitigation ambition, TXx increases by more than 1°C over the studied cities. To put it another way, TXx over the cities would drop more than the global mean temperature reduction if international mitigation ambition is increased from the current NDCs to levels that meet the Paris Agreement goal. Reducing future global mean temperature rise from 3° to 2°C would reduce TXx over the cities by 1.1° to 1.8°C, whereas reducing temperature rise from 3° to 1.5°C would reduce TXx over the cities by 1.6° to 2.8°C. This means that meeting the Paris Agreement long-term temperature goal could significantly and effectively reduce exposure to extreme heat in the studied U.S. cities.

### Temperature-mortality relationships

To quantify the extent to which decreased exposure to extreme heat could decrease the associated heat-related mortality, we determined the exposure-response relationship between daily mean temperature and mortality for each city using observed data from 1987 to 2000 (see Materials and Methods). Consistent with the literature, the temperature-mortality relationships have a common U or J shape but are location specific (fig. S1). The minimum mortality temperature (MMT; dotted line on fig. S1) is where the risk of heat-related mortality is lowest. Here, the MMT is defined as the minimum mortality percentile between the 2nd and 98th percentiles of the observed temperature range over each city [cf. Gasparrini *et al.* (*5*)]. This minimum mortality percentile lies between the 80th and 90th percentiles for nine of the studied cities and between the 30th and ~60th percentiles for some of the subtropical cities such as Miami and Houston (table S1). The corresponding MMT ranges from 15°C in St. Louis to 34.5°C in Phoenix. The different temperature-mortality relationships and MMTs across the cities highlight the importance of city-specific analyses such as this one.

Temperatures above and below the MMT are associated with elevated mortality risks (except for extreme heat-related mortality in Atlanta and San Francisco; fig. S1, A and L). We express mortality risk as the overall cumulative relative risk (RR) (*5*), i.e., the cumulated mortality risk over a period of 21 days relative to that at the MMT (see Materials and Methods). We find higher RR to be associated with extreme heat (defined as the 99th temperature percentile) for northern cities such as Boston, Detroit, New York City, and Philadelphia than for southern cities such as Houston, Miami, and Phoenix. Nevertheless, the heat part of the temperature-mortality relationships for the southern cities is highly uncertain (red line and gray shading on fig. S1). The north-south difference in heat RR demonstrates that the temperature-mortality relationships are, in part, latitude dependent, although various contextual factors including demographic characteristics (*34*), air pollution, and socioeconomic status (*35*–*37*) are also known to affect a location’s vulnerability to heat. For Atlanta and San Francisco, RR drops below 1 (albeit with large uncertainty) at temperatures above ~30° and ~27°C, respectively. This might be attributed to unstable exposure-response relationships resulting from few extreme hot days in the observations. We assume that the temperature-mortality relationship and MMT for a given city do not change with increasing regional temperatures. We discuss the potential limitations of this assumption in Discussion.

### Avoidable fraction of mortality attributable to heat

**Fig. 2:**
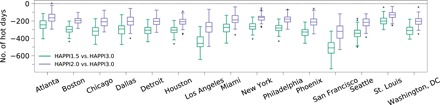
Differences in the number of hot days over a decade-long period between the 1.5° and 2°C scenarios and the 3°C baseline scenario over the studied U.S. cities. The box plots show the distribution of differences computed from 90 ensemble members, with the middle line showing the median, the box showing the interquartile range (IQR), the whiskers indicating values that are 1.5 IQR from the lower and upper quartiles, and the dots indicating any outliers. A box plot that does not include 0 indicates a significant difference in the number of hot days between the two scenarios.

For each studied city, we define days on which mean temperature falls above the MMT as “hot days” (see Discussion). We counted the number of hot days in each decade-long HAPPI simulation (see Materials and Methods). If the 2°C warmer world is realized rather than the 3°C world, then all studied cities would experience significantly fewer hot days (Fig. 2). The median reduction in the number of hot days ranges from 129 days per decade in St. Louis to 319 days per decade in San Francisco. This means that nearly 10% of days would turn from warmer than the MMT to cooler than the MMT in San Francisco in the case of 2°C warming above preindustrial levels instead of 3°C.

By meeting the 1.5°C threshold rather than 3°C, all studied cities could experience a significant reduction in the number of hot days. The median reduction across the ensemble members ranges from 202 days per decade in St. Louis to 510 days per decade in San Francisco. This means that the equivalent of 1.3 in 10 years would turn from warmer than the MMT to cooler than the MMT in San Francisco if the stricter Paris Agreement threshold is achieved.

The projected number of hot days under 1.5°C warming is consistently lower than that under 2°C warming for all cities. This highlights the increased benefit of ratcheting up mitigation ambition to meet the 1.5°C Paris Agreement threshold in terms of the overall heat exposure of the studied cities.

In each experiment, for each day over each city, we used the overall cumulative RR corresponding to that city and that day’s temperature to calculate the number of deaths attributable to temperature anomalies from the corresponding MMT (*5*, *38*) (see Materials and Methods). We divided the sum of attributable deaths on all hot days by the total number of all-cause deaths expected in a decade to obtain the attributable fraction of heat-related mortality. The difference in attributable fraction of heat-related mortality between the 3°C experiment and other HAPPI experiments gives the avoidable attributable fraction.

**Fig. 3:**
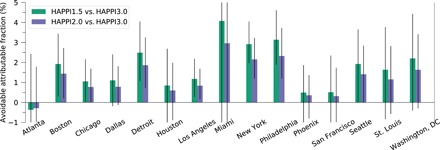
Avoidable fraction of heat-related deaths if the current trajectory warming of 3°C is brought down to the 1.5° or 2°C Paris Agreement thresholds. The value of each bar indicates the mean avoidable attributable fraction across 90 climate model ensemble members. The error bars show the 95% eCI that accounts for both the uncertainty arising from internal climate variability and the uncertainty associated with the estimated exposure-response relationship (gray shading in fig. S1). Confidence intervals that do not include 0 indicate statistically significant results at the 5% level.

Figure 3 shows the avoidable attributable fractions of heat-related mortality in the studied U.S. cities, if 3°C warming is reduced to the Paris Agreement thresholds. If global warming is limited to 2°C instead of 3°C above preindustrial levels, then heat-related mortality would be reduced in all of the cities studied, except for Atlanta, which had an unstable exposure-response relationship (see section above). In particular, Boston, Chicago, Detroit, Los Angeles, New York City, Philadelphia, and Seattle would see a significant attributable fraction of avoided heat-related mortality [ranging from 0.8%; 95% empirical confidence interval (eCI), 0.03 to 1.7% in Chicago to 2.3%; 95% eCI, 1.2 to 3.7% in Philadelphia] if the 2°C threshold is met as compared with the 3°C trajectory.

In a 1.5°C warmer world, all cities except Atlanta would have avoided heat-related mortality relative to the 3°C world. The avoidable attributable fraction is larger in all of these cities in the 1.5°C than the 2°C warmer world. Boston, Chicago, Detroit, Los Angeles, New York City, Philadelphia, and Seattle could still see a significant fraction of avoided heat-related mortality. The locations with statistically significant effects range from 1.1% (95% eCI, 0.05 to 2.2%) in Chicago to 3.1% (95% eCI, 1.9 to 4.6%) in Philadelphia. This suggests substantial benefits (in terms of reduced heat-related mortality) of increasing international mitigation ambition to achieve the stricter Paris Agreement threshold. Echoing the findings of Mitchell *et al.* (*18*) who looked at Europe and Vicedo-Cabrera *et al.* (*20*) who looked at country-level mortality, our results demonstrate that reducing future temperature rise by that extra half a degree between the two Paris Agreement thresholds could provide substantial benefits with respect to heat-related mortality in the studied U.S cities.

### Avoidable 1-in-30-year heat-related mortality

Modeling climate in the HAPPI scenarios with 90 ensemble members allows us to not only separate anthropogenic signals from internal variability but also isolate policy-relevant return periods of major extreme heat events. Using the annual numbers of projected heat-related deaths over the 900 years of simulation for each HAPPI experiment (see Materials and Methods), we compared the 1-in-30-year heat-related mortality level between HAPPI3.0 and the Paris Agreement thresholds individually. We assume constant population at the 1987–2000 levels throughout this study (see Discussion).

**Fig. 4:**
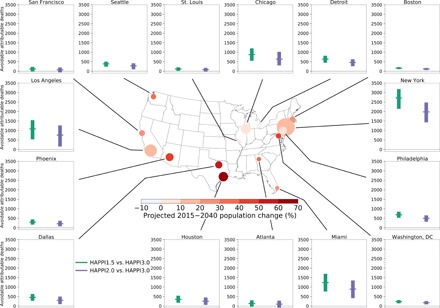
One-in-30-year heat-related mortality that is avoidable by stabilizing future warming at the 1.5° and 2°C Paris Agreement thresholds rather than 3°C. The point estimates show the mean 1-in-30-year mortality level across 101 plausible exposure-response relationships, whereas the error bars show the 95% eCI accounting for uncertainties from internal climate variability and the exposure-response relationship. All estimates assume constant population. Confidence intervals that do not include 0 (dotted line on each panel) indicate a statistically significant number of avoidable deaths. The size of each bubble on the central map is proportional to the square root of the city’s population in July 2016. The color of each bubble indicates the city’s projected population change between 2015 and 2040. Other return periods are given in fig. S3.

Figure 4 shows the heat-related mortality associated with a 1-in-30-year event that could be avoided if international mitigation ambition is increased to reduce 3°C warming to 2° or 1.5°C. On average, limiting warming to 2°C avoids between 75 and 1980 heat-related deaths once every 30 years depending on the city. In 12 of the cities (all excluding Atlanta, San Francisco, and St. Louis), the number of 1-in-30-year heat-related deaths avoided is statistically significant (at the 5% level) if the 2°C warmer world is realized rather than the 3°C one. By further limiting warming to 1.5°C, the 15 studied cities would have an average of between 114 and 2716 heat-related deaths avoided once every 30 years. For 13 of these cities (i.e., excluding Atlanta and San Francisco), these avoided 1-in-30-year heat-related deaths are statistically significant at the 5% significance level.

New York City, the most populous city in the United States (as indicated by the size of the bubbles on Fig. 4), could see 1980 (95% eCI, 1504 to 2475) 1-in-30-year heat-related deaths avoided in the 2°C warmer world relative to the 3°C warmer world under the assumption of constant population. If the 1.5°C world is realized, then 2716 (95% eCI, 2138 to 3181) of 1-in-30-year heat-related deaths could be avoided, relative to 3°C.

Los Angeles, the second most populous U.S. city, is projected to have 759 (95% eCI, 167 to 1265) 1-in-30-year avoided heat-related deaths under the 2°C threshold, relative to 3°C. Under the 1.5°C threshold, 1085 (95% eCI, 534 to 1543) 1-in-30-year heat-related deaths could be avoided. Significant numbers of 1-in-30-year heat-related deaths could be avoided in New York City and Los Angeles under either of the Paris Agreement thresholds, with hundreds more heat-related deaths avoided in each city by limiting warming to 1.5°C. Achieving the stricter Paris Agreement threshold is therefore crucial to reducing policy-relevant heat-related mortality levels in the most populous U.S. cities. The avoidable 1-in-30-year heat-related deaths per 100,000 persons can be found in fig. S2.

Chicago is one of the 12 cities that could experience a statistically significant reduction in 1-in-30-year heat-related mortality relative to the 3°C warmer world under both Paris Agreement thresholds. Specifically, 636 (95% eCI, 303 to 999) and 875 (95% eCI, 546 to 1206) 1-in-30-year heat-related deaths could be avoided in the 2° and 1.5°C warmer world, under an assumption of constant population. To put these numbers into context, the July 1995 Chicago heat wave led to 514 excess heat-related deaths in the city (*39*). This means that increasing mitigation ambition to meet the 2° and 1.5°C Paris Agreement thresholds could respectively avoid 124 and 170% of the deaths caused by the 1995 heat wave, once every 30 years.

The historic 1995-like heat-related mortality event in Chicago could change from a 1-in-1.4-year event (eCI, 1.3 to 1.7 years) under the 3°C trajectory to a 1-in-2.8-year event (eCI, 2.2 to 3.5 years) in the 2°C world and an even less frequent 1-in-4.7-year event (eCI, 3.7 to 6.6 years) in a 1.5°C world (fig. S3). These return periods highlight the importance of strengthening efforts toward achieving the Paris Agreement long-term temperature goal, especially the 1.5°C threshold, to reduce the recurrence probability of a deadly historical heat event compared to the climate we may expect, consistent with current mitigation ambition. Full return period curves can be found in figs. S3 and S4.

## DISCUSSION

Our analyses focused on the heat-related mortality avoided from lowering our current emission pathways to those consistent with the Paris Agreement thresholds. This study uses a large initial condition ensemble to assess uncertainty due to internal climate variability versus greenhouse gas forced changes and to compute return periods of extreme mortality events. Like all extreme event attribution studies, we discerned the heat-health benefits of additional mitigation by keeping all other conditions—including constant population, temperature-mortality relationships, and MMTs—in 1987–2000 between different scenarios (*40*).

In reality, however, the population of a city is ever-changing. Population size of the studied U.S. cities have changed since 1987 and are projected to change by −3.5% in Detroit to +66.7% in Houston between 2015 and 2040 (Fig. 4) (*41*). The population structure of the cities is likely to change too. For example, 15.2% of the national population is 65 years old or above (July 2016 estimates) (*42*). This proportion is projected to rise to 22% in 2040 (*43*). Older adults are more susceptible to heat-related mortality than other age groups, with those aged 85 or above most at risk of heat-related mortality (*39*, *44*). Therefore, population increase and an aging population, along with changes in other demographic characteristics that contribute to heat vulnerability (*34*) and increases in urbanization, will likely exacerbate heat-related mortality in the coming years. This will be compounded by higher levels of warming associated with urban heat island effects (depending on urbanization choices). Thus, the avoidable deaths projected in this study may be conservative estimates, reinforcing the benefits of ratcheting up mitigation ambition to prevent elevation of heat-related mortality in the United States.

Municipal adaptation and acclimatization may reduce future heat-related mortality. Increased availability of air conditioning, awareness of heat-related health risks, and improved health care reduced heat-related mortality in the United States over the last few decades (*38*, *45*–*48*). According to the 2015 American Housing Survey (*49*) and Residential Energy Consumption Survey (*50*), current prevalence of air conditioning ranges from 34% in Seattle to more than 99% in Atlanta and Houston. This underscores the important role of municipal adaptation strategies in increasing resilience to future higher temperatures. Acclimatization could also alter temperature-mortality relationships, lowering the projected heat-related mortality and adding uncertainty to estimated avoidable mortality under the Paris Agreement thresholds.

We used MMTs estimated over the period 1987–2000 as reference temperatures for estimating heat-related mortality. We assumed a constant MMT for a location so that we could attribute changes in climate rather than project changes based on assumed adaptation, as is commonplace in temperature-mortality studies (*7*, *9*, *13*, *20*). We note that a particular location can be associated with a range of MMTs due to differences in modeling choices (*51*) and the nature of the temperature-mortality relationship (*52*). Because of acclimatization, adaptation, and demographic changes, the MMTs for Stockholm (*53*) and Japan (*54*) both increased over the past decades. As the population acclimatizes to warmer ambient temperatures and as other factors such as access to air conditioning change, the MMTs for the studied U.S. cities may change over time, altering our mortality estimates.

We simultaneously assessed the nonlinear relationship between temperature and mortality over the whole range of observed temperatures in each city, including any delayed effects of up to 21 days of lag. Previous studies that took the same approach estimated the mortality impacts of both heat and cold (*13*, *15*, *20*). We did not estimate cold-related mortality changes or compute the combined changes in heat- and cold-related mortality because extreme high and low temperatures have different exposure-response relationships, including different lag structures, which we did not fully account for in this study. High ambient temperatures have an immediate and direct effect on mortality. Associations between low ambient temperatures and different causes of mortality are less direct, with longer lags (*55*, *56*). Therefore, heat- and cold-related mortality should be studied separately. As different adaptation strategies are also needed to offset heat and cold health impacts, future work is needed to specifically model the relationship between low ambient temperatures and mortality and to look at how cold-related mortality may differ between 3°C warming and the Paris Agreement long-term temperature thresholds.

## CONCLUSIONS

Recognizing the threat of climate change, the Paris Agreement has been ratified by 181 parties to limit global temperature rise this century to well below 2°C or even 1.5°C above preindustrial levels. Current mitigation ambition as established in the initial NDCs implies ~3°C warming. We asked whether and to what extent putting additional mitigation effort into reducing future warming from 3° to 2°C or 1.5°C would avoid heat-related mortality over 15 U.S. cities.

The answers to this question are clear: (i) Ratcheting up global mitigation ambition to achieve the Paris Agreement long-term temperature goal would significantly reduce these cities’ exposure to extreme heat, (ii) limiting temperature rise to 2°C above preindustrial levels would reduce the fraction of mortality attributable to heat and the 1-in-30-year heat-related mortality over most studied U.S. cities, and (iii) limiting temperature rise to 1.5°C above preindustrial levels would be substantially more beneficial allowing the temperature to reach 2°C. Our results demonstrate that strengthened mitigation ambition would result in substantial benefits to public health in the United States.

## MATERIALS AND METHODS

### Exposure-response modeling

We obtained daily counts of all-cause deaths and daily mean temperatures for the 1987–2000 period for 15 U.S. cities (Atlanta, Boston, Chicago, Dallas, Detroit, Houston, Los Angeles, Miami, New York City, Philadelphia, Phoenix, San Francisco, Seattle, St. Louis, and Washington, DC) from the NMMAPS (*31*). We statistically modeled the delayed and nonlinear relationship between daily mean temperature and all-cause mortality over the whole range of observed temperatures for each city using distributed lag nonlinear models (*5*, *57*). This type of model simultaneously describes the exposure-response relationship and an additional lag-response relationship. We considered a lag period of up to 21 days to capture any delayed responses, as was done in previous studies (*5*, *6*).

We used a natural cubic spline with the same internal knots as that used in Gasparrini *et al.* (*13*) to model the exposure-response curve for each city. This allows for log-linear extrapolation of the temperature-mortality relationship beyond the observed temperature range. Extrapolation is essential for higher temperatures projected under climate change (see table S2 for specific details). We acknowledge that the log-linear extrapolation applied may be conservative in estimating the true exposure-response relationship at temperatures higher than the observed range (*58*).

Nevertheless, the temperature-mortality relationships we found were robust to the choice of observational period and spline model. Gasparrini *et al.* (*5*) used a longer (1985–2006) dataset and a quadratic B-spline model and found similar relationships for the same cities. The 21-day lag period that we used was also sufficient to capture potential delayed responses, as our relationships were comparable to those found by Anderson and Bell (*6*) using a longer lag period (28 days). Furthermore, Curriero *et al.* (*59*) reported higher heat-related relative mortality risks over northeastern U.S. cities versus southeastern cities between 1973 and 1994, a characteristic that we found with our dataset and statistical model.

### Climate modeling

To account for different internal climate variability states, the decade-long HAPPI time-slice experiments were uniquely set up with 90 ensemble members. The large ensembles allow for the study of extremes in specifically designed Paris Agreement scenarios. We used the 90-member ensemble of daily mean temperature simulations from the atmosphere-only general circulation model HadAM3P (*29*, *30*). The ensemble members were initialized by applying perturbations to the potential temperature field of the atmosphere taken from consecutive 1-day difference fields in a long run of the model. HadAM3P has a horizontal latitude-longitude resolution of 1.25 × 1.875° (N96) (*30*, *60*). Through the citizen science weather@home project (*30*, *60*), we followed the HAPPI protocol (*61*) and used the 1.5° and 2°C time-slice experiments along with a new 3°C experiment. These experiments represent stabilized climates at 1.5°, 2°, and 3°C above preindustrial levels in any 10-year period in the future.

The HAPPI methodology (*61*) for determining the 2°C world calculated sea surface temperatures, sea ice concentrations, and the radiative forcing of well-mixed greenhouse gases by using a weighted sum of Representative Concentration Pathways (RPC) from the Coupled Model Intercomparison Project Phase 5 (CMIP5) (*62*). We used the same approach for the HAPPI 3°C experiment, but with different weighting. We took the weighted sum of the RCP4.5 and RCP8.5 scenarios in the form of 0.686 × RCP4.5 + 0.314 × RCP8.5. These weights were calculated such that the CMIP5 multi-model mean global mean temperature over 2091–2100 was ~3°C above the 1861–1880 level.

For estimation of future mortality in the cities of interest, we extracted the bias-corrected daily mean temperature time series (see below) over the land grid cells that were geographically closest to the individual cities.

### Bias correction

We bias-corrected the daily mean and maximum temperature output of the HAPPI experiments against observations using the Inter-Sectoral Impact Model Intercomparison Project (ISI-MIP) bias correction approach (*63*). We linearly interpolated the temperature output from the HadAM3P 1987–2016 simulations (in a 360-day calendar) to the standard Gregorian calendar for comparison with observations. For Fig. 1 (B to D), we followed the newest ISI-MIP protocol (*27*) and used the ﻿EWEMBI dataset (1979–2013 daily data at 0.5 × 0.5° horizontal resolution) (*27*, *28*) as reference for bias correction. Specifically, we interpolated the EWEMBI data onto the N96 grid and used the common period between the HadAM3P 1987–2016 simulations and EWEMBI, i.e., 1987–2013, as the reference period for computing the bias correction factors (see below). For the rest of this study, we used the 1987–2000 NMMAPS daily mean temperature observations as reference for bias-correcting the HAPPI daily mean temperature simulations in the 15 U.S. cities in this study.

For each calendar month and over each grid cell, we added a constant offset that equaled the average difference between the observed and simulated monthly mean data over the reference period to the time series of simulated temperatures in the HAPPI experiments. To correct the daily variability in the HAPPI simulations, we adjusted the distribution of the daily temperature residuals (around the corresponding monthly means) in the HAPPI simulations to that of the observations through a transfer function. This transfer function was derived from linear regression between the ranked observed residuals and simulated residuals over the reference period. Please refer to Hempel *et al.* (*63*) for specific details. All in all, by doing so, we preserved the long-term absolute trend in the simulations while correcting the daily variability to that of the observations.

### Computation of avoidable mortality and its uncertainty

We quantified the uncertainty associated with exposure-response modeling by using 100 plausible temperature-mortality relationships in addition to the mean relationship (blue and red lines in fig. S1) for each city. We generated these samples using Monte Carlo simulations, assuming a multivariate normal distribution of the spline model coefficients (*5*, *38*). For each decade-long simulation in the 90-member climate model ensemble, we used all 101 temperature-mortality relationships to estimate the number of heat-related deaths.

For Fig. 3, this means that we computed 9090 (90 ensemble members × 101 temperature-mortality relationships) fractions of mortality attributable to heat for each city and climate scenario. We computed the avoidable attributable fractions of heat-related mortality by subtracting the attributable fraction in HAPPI1.5 or HAPPI2.0 from the corresponding attributable fraction in HAPPI3.0. In other words, the avoidable fractions are paired differences. We took the mean avoidable fraction across the 90 ensemble members (considering only the mean temperature-mortality relationship) as the point estimate and the 2.5 to 97.5 percentiles of all 9090 estimates of attributable fractions as the 95% eCI on Fig. 3.

For Fig. 4 and figs. S2 to S4, we used the 900 model years in each experiment (10 years of simulation × 90 ensemble members) to estimate mortality levels of rare events. Using each of the 101 plausible temperature-mortality relationships, we determined the annual numbers of heat-related deaths in the 900-year series and sorted them in descending order. For figs. S2 and S4, we took the additional step of dividing the series by the city’s July 2016 population in 100,000 persons. The return periods of these mortality levels were then 900 divided by their ranks within the sorted series. This means that the highest mortality level is always the rarest (a 1-in-900-year event). Using the 101 plausible temperature-mortality relationships, we generated 101 return period curves for each climate scenario. Each solid line in figs. S3 and S4 represents the mean return period curve across the 101 estimates. We estimated the 95% confidence interval associated with climate variability by bootstrapping this mean curve 1000 times. We added this uncertainty to the 2.5 to 97.5 percentiles of all 101 curves, generating a combined 95% eCI, as indicated by the shading in figs. S3 and S4. We subtracted the 1-in-30-year mortality level in HAPPI1.5 or HAPPI2.0 from HAPPI3.0 for Fig. 4 and fig. S2. Increasing the temperature-mortality relationship sample size from 100 to 500 did not alter the main results.

## Supplementary Material

http://advances.sciencemag.org/cgi/content/full/5/6/eaau4373/DC1

Download PDF

## SUPPLEMENTARY MATERIALS

Supplementary material for this article is available at http://advances.sciencemag.org/cgi/content/full/5/6/eaau4373/DC1 Fig. S1. Estimated exposure-response relationships between daily mean temperature and all-cause mortality over selected U.S. cities. Fig. S2. One-in-30-year heat-related mortality per 100,000 persons that is avoidable by stabilizing future warming at the 1.5° and 2°C Paris Agreement thresholds rather than 3°C. Fig. S3. Heat-related mortality return period curves in future stabilization scenarios of 1.5°, 2°, and 3°C. Fig. S4. Population-normalized heat-related mortality return period curves in future stabilization scenarios of 1.5°, 2°, and 3°C. Table S1. The MMT and its percentile rank in the 1987–2000 observations in each city. Table S2. Maximum observed and projected temperatures and the percentage of days on which the projected temperature exceeds the maximum observed temperature in each scenario and city.

## References

[1] Rogelj J., den Elzen M., Höhne N., Fransen T., Fekete H., Winkler H., Schaeffer R., Sha F., Riahi K., Meinshausen M., Paris Agreement climate proposals need a boost to keep warming well below 2 °C. Nature 534, 631–639 (2016). 27357792 10.1038/nature18307

[2] UNFCCC, *Adoption of the Paris Agreement. Proposal by the President, Conference of the Parties, Twenty-first session*, Paris, 30 November to 11 December 2015 (UNFCCC, 2015).

[3] Intergovernmental Panel on Climate Change, Summary for Policymakers in *Global Warming of 1.5°C. An IPCC Special Report on the impacts of global warming of 1.5°C above pre-industrial levels and related global greenhouse gas emission pathways, in the context of strengthening the global response to the threat of climate change, sustainable development, and efforts to eradicate poverty* (IPCC, 2018), pp. 1–33; http://report.ipcc.ch/sr15/pdf/sr15_spm_final.pdf.

[4] T. Fransen, E. Northrop, “4 reasons for countries to enhance their NDCs by 2020,” 07 November 2017; www.wri.org/blog/2017/11/4-reasons-countries-enhance-their-ndcs-2020.

[5] Gasparrini A., Guo Y., Hashizume M., Lavigne E., Zanobetti A., Schwartz J., Tobias A., Tong S., Rocklöv J., Forsberg B., Leone M., De Sario M., Bell M. L., Guo Y. L. L., Wu C. F., Kan H., Yi S. M., de Sousa Zanotti Stagliorio Coelho M., Saldiva P. H. N., Honda Y., Kim H., Armstrong B., Mortality risk attributable to high and low ambient temperature: A multicountry observational study. Lancet 386, 369–375 (2015). 26003380 10.1016/S0140-6736(14)62114-0 PMC4521077

[6] Anderson B. G., Bell M. L., Weather-related mortality: How heat, cold, and heat waves affect mortality in the United States. Epidemiology 20, 205–213 (2012). 10.1097/EDE.0b013e318190ee08 PMC3366558 19194300

[7] Ma W., Chen R., Kan H., Temperature-related mortality in 17 large Chinese cities: How heat and cold affect mortality in China. Environ. Res. 134, 127–133 (2014). 25127523 10.1016/j.envres.2014.07.007

[8] Guo Y., Gasparrini A., Armstrong B., Li S., Tawatsupa B., Tobias A., Lavigne E., de Sousa Zanotti Stagliorio Coelho M., Leone M., Pan X., Tong S., Tian L., Kim H., Hashizume M., Honda Y., Guo Y. L. L., Wu C. F., Punnasiri K., Yi S. M., Michelozzi P., Saldiva P. H., Williams G., Global variation in the effects of ambient temperature on mortality: A systematic evaluation. Epidemiology 25, 781–789 (2014). 25166878 10.1097/EDE.0000000000000165 PMC4180721

[9] Weinberger K. R., Haykin L., Eliot M. N., Schwartz J. D., Gasparrini A., Wellenius G. A., Projected temperature-related deaths in ten large U.S. metropolitan areas under different climate change scenarios. Environ. Int. 107, 196–204 (2017). 28750225 10.1016/j.envint.2017.07.006 PMC5575805

[10] Kingsley S. L., Eliot M. N., Gold J., Vanderslice R. R., Wellenius G. A., Current and projected heat-related morbidity and mortality in Rhode Island. Environ. Health Perspect. 124, 460–467 (2016). 26251954 10.1289/ehp.1408826 PMC4829994

[11] Ostro B., Barrera-Gómez J., Ballester J., Basagaña X., Sunyer J., The impact of future summer temperature on public health in Barcelona and Catalonia, Spain. Int. J. Biometeorol. 56, 1135–1144 (2012). 22370738 10.1007/s00484-012-0529-7

[12] Huynen M. M. T. E., Martens P., Climate change effects on heat- and cold-related mortality in the Netherlands: A scenario-based integrated environmental health impact assessment. Int. J. Environ. Res. Public Health 12, 13295–13320 (2015). 26512680 10.3390/ijerph121013295 PMC4627032

[13] Gasparrini A., Guo Y., Sera F., Vicedo-Cabrera A. M., Huber V., Tong S., de Sousa Zanotti Stagliorio Coelho M., Saldiva P. H. N., Lavigne E., Correa P. M., Ortega N. V., Kan H., Osorio S., Kyselý J., Urban A., Jaakkola J. J. K., Ryti N. R. I., Pascal M., Goodman P. G., Zeka A., Michelozzi P., Scortichini M., Hashizume M., Honda Y., Hurtado-Diaz M., Cruz J. C., Seposo X., Kim H., Tobias A., Iñiguez C., Forsberg B., Åström D. O., Ragettli M. S., Guo Y. L., Wu C. F., Zanobetti A., Schwartz J., Bell M. L., Dang T. N., Van D. D., Heaviside C., Vardoulakis S., Hajat S., Haines A., Armstrong B., Projections of temperature-related excess mortality under climate change scenarios. Lancet Planet. Health 1, e360–e367 (2017). 29276803 10.1016/S2542-5196(17)30156-0 PMC5729020

[14] Chen K., Horton R. M., Bader D. A., Lesk C., Jiang L., Jones B., Zhou L., Chen X., Bi J., Kinney P. L., Impact of climate change on heat-related mortality in Jiangsu Province, China. Environ. Pollut. 224, 317–325 (2017). 28237309 10.1016/j.envpol.2017.02.011 PMC5387110

[15] Guo Y., Li S., Liu D. L., Chen D., Williams G., Tong S., Projecting future temperature-related mortality in three largest Australian cities. Environ. Pollut. 208, 66–73 (2016). 26475058 10.1016/j.envpol.2015.09.041

[16] Ahmadalipour A., Moradkhani H., Escalating heat-stress mortality risk due to global warming in the Middle East and North Africa (MENA). Environ. Int. 117, 215–225 (2018). 29763817 10.1016/j.envint.2018.05.014

[17] King A. D., Donat M. G., Lewis S. C., Henley B. J., Mitchell D. M., Stott P. A., Fischer E. M., Karoly D. J., Reduced heat exposure by limiting global warming to 1.5 °C. Nat. Clim. Change 8, 549–551 (2018).

[18] Mitchell D., Heaviside C., Schaller N., Allen M., Ebi K. L., Fischer E. M., Gasparrini A., Harrington L., Kharin V., Shiogama H., Sillmann J., Sippel S., Vardoulakis S., Extreme heat-related mortality avoided under Paris Agreement goals. Nat. Clim. Change 8, 551–553 (2018). 10.1038/s41558-018-0210-1 PMC6181199 30319715

[19] Sylla M. B., Faye A., Giorgi F., Diedhiou A., Kunstmann H., Projected heat stress under 1.5 °C and 2 °C global warming scenarios creates unprecedented discomfort for humans in West Africa. Earths Future 6, 1029–1044 (2018).

[20] Vicedo-Cabrera A. M., Guo Y., Sera F., Huber V., Schleussner C.-F., Mitchell D., Tong S., de Sousa Zanotti Stagliorio Coelho M., Saldiva P. H. N., Lavigne E., Matus P., Correa N. V. O., Kan H., Osorio S., Kyselý J., Urban A., Jaakkola J. J. K., Ryti N. R. I., Pascal M., Goodman P. G., Zeka A., Michelozzi P., Scortichini M., Hashizume M., Honda Y., Hurtado-Diaz M., Cruz J., Seposo X., Kim H., Tobias A., Íñiguez C., Forsberg B., Åström D. O., Ragettli M. S., Röösli M., Guo Y. L., Wu C. F., Zanobetti A., Schwartz J., Bell M. L., Dang T. N., Van D. D., Heaviside C., Vardoulakis S., Hajat S., Haines A., Armstrong B., Ebi K. L., Gasparrini A., Temperature-related mortality impacts under and beyond Paris Agreement climate change scenarios. Clim. Change 150, 391–402 (2018). 30405277 10.1007/s10584-018-2274-3 PMC6217994

[21] Mitchell D., James R., Forster P. M., Betts R. A., Shiogama H., Allen M., Realizing the impacts of a 1.5 °C warmer world. Nat. Clim. Change 6, 735–737 (2016).

[22] Jeffery M. L., Gütschow J., Rocha M. R., Gieseke R., Measuring success: Improving assessments of aggregate greenhouse gas emissions reduction goals. Earths Future 6, 1260–1274 (2018).

[23] Kottek M., Grieser J., Beck C., Rudolf B., Rubel F., World map of the Köppen-Geiger climate classification updated. Meteorol. Z. 15, 259–263 (2006).

[24] Sillmann J., Donat M. G., Fyfe J. C., Zwiers F. W., Observed and simulated temperature extremes during the recent warming hiatus. Environ. Res. Lett. 9, 064023 (2014).

[25] Seneviratne S. I., Donat M. G., Pitman A. J., Knutti R., Wilby R. L., Allowable CO_2_ emissions based on regional and impact-related climate targets. Nature 529, 477–483 (2016). 26789252 10.1038/nature16542

[26] Sillmann J., Kharin V. V., Zhang X., Zwiers F. W., Bronaugh D., Climate extremes indices in the CMIP5 multimodel ensemble: Part 1. Model evaluation in the present climate. J. Geophys. Res. Atmos. 118, 1716–1733 (2013).

[27] Frieler K., Lange S., Piontek F., Reyer C. P. O., Schewe J., Warszawski L., Zhao F., Chini L., Denvil S., Emanuel K., Geiger T., Halladay K., Hurtt G., Mengel M., Murakami D., Ostberg S., Popp A., Riva R., Stevanovic M., Suzuki T., Volkholz J., Burke E., Ciais P., Ebi K., Eddy T. D., Elliott J., Galbraith E., Gosling S. N., Hattermann F., Hickler T., Hinkel J., Hof C., Huber V., Jägermeyr J., Krysanova V., Marcé R., Müller Schmied H., Mouratiadou I., Pierson D., Tittensor D. P., Vautard R., van Vliet M., Biber M. F., Betts R. A., Bodirsky B. L., Deryng D., Frolking S., Jones C. D., Lotze H. K., Lotze-Campen H., Sahajpal R., Thonicke K., Tian H., Yamagata Y., Assessing the impacts of 1.5°C global warming—Simulation protocol of the Inter-Sectoral Impact Model Intercomparison Project (ISIMIP2b). Geosci. Model Dev. 10, 4321–4345 (2017).

[28] Lange S., Bias correction of surface downwelling longwave and shortwave radiation for the EWEMBI dataset. Earth Syst. Dynam. 9, 627–645 (2018).

[29] Pope V. D., Gallani M. L., Rowntree P. R., Stratton R. A., The impact of new physical parametrizations in the Hadley Centre climate model: HadAM3. Climate Dynam. 16, 123–146 (2000).

[30] Guillod B. P., Jones R. G., Bowery A., Haustein K., Massey N. R., Mitchell D. M., Otto F. E. L., Sparrow S. N., Uhe P., Wallom D. C. H., Wilson S., Allen M. R., weather@home 2: Validation of an improved global-regional climate modelling system. Geosci. Model Dev. 10, 1849–1872 (2017).

[31] Samet J. M., Dominici F., Zeger S. L., Schwartz J., Dockery D. W., The National morbidity, mortality, and air pollution study. Part I: Methods and methodologic issues. Res. Rep. Health Eff. Inst. 94, 5-14; discussion 75–84 (2000). 11098531

[32] Wartenburger R., Hirschi M., Donat M. G., Greve P., Pitman A. J., Seneviratne S. I., Changes in regional climate extremes as a function of global mean temperature: An interactive plotting framework. Geosci. Model Dev. 10, 3609–3634 (2017).

[33] Wang Z., Lin L., Zhang X., Zhang H., Liu L., Xu Y., Scenario dependence of future changes in climate extremes under 1.5 °C and 2 °C global warming. Sci. Rep. 7, 1–9 46432 (2017). 28425445 10.1038/srep46432 PMC5397837

[34] Reid C. E., O’Neill M. S., Gronlund C. J., Brines S. J., Brown D. G., Diez-Roux A. V., Schwartz J., Mapping community determinants of heat vulnerability. Environ. Health Perspect. 117, 1730–1736 (2009). 20049125 10.1289/ehp.0900683 PMC2801183

[35] Sera F., Armstrong B., Tobias A., Vicedo-Cabrera A. M., Åström C., Bell M. L., Chen B.-Y., de Sousa Zanotti Stagliorio Coelho M., Matus Correa P., Cruz J. C., Dang T. N., Hurtado-Diaz M., Do Van D., Forsberg B., Guo Y. L., Guo Y., Hashizume M., Honda Y., Iñiguez C., Jaakkola J. J. K., Kan H., Kim H., Lavigne E., Michelozzi P., Ortega N. V., Osorio S., Pascal M., Ragettli M. S., Ryti N. R. I., Saldiva P. H. N., Schwartz J., Scortichini M., Seposo X., Tong S., Zanobetti A., Gasparrini A., How urban characteristics affect vulnerability to heat and cold: A multi-country analysis. Int. J. Epidemiol. 0, 1–12 (2019). 10.1093/ije/dyz008 30815699

[36] Benmarhnia T., Oulhote Y., Petit C., Lapostolle A., Chauvin P., Zmirou-Navier D., Deguen S., Chronic air pollution and social deprivation as modifiers of the association between high temperature and daily mortality. Environ. Health 13, 53 (2014). 24941876 10.1186/1476-069X-13-53 PMC4073194

[37] Huang Z., Lin H., Liu Y., Zhou M., Liu T., Xiao J., Zeng W., Li X., Zhang Y., Ebi K. L., Tong S., Ma W., Wang L., Individual-level and community-level effect modifiers of the temperature–mortality relationship in 66 Chinese communities. BMJ Open 5, e009172 (2015). 10.1136/bmjopen-2015-009172 PMC4577931 26369803

[38] Gasparrini A., Leone M., Attributable risk from distributed lag models. BMC Med. Res. Methodol. 14, 55 (2014). 24758509 10.1186/1471-2288-14-55 PMC4021419

[39] Whitman S., Good G., Donoghue E. R., Benbow N., Shou W., Mou S., Mortality in Chicago attributed to the July 1995 heat wave. Am. J. Public Health 87, 1515–1518 (1997). 9314806 10.2105/ajph.87.9.1515 PMC1380980

[40] Stott P. A., Karoly D. J., Zwiers F. W., Is the choice of statistical paradigm critical in extreme event attribution studies? Clim. Change 144, 143–150 (2017).

[41] G. S. Thomas, “ACBJ’s population projections for 933 U.S. markets through 2040,” 11 October 2016; www.bizjournals.com/bizjournals/news/2016/10/11/projectionsdatabase.html.

[42] United States Census Bureau, QuickFacts United States, 2017; www.census.gov/quickfacts.

[43] United States Census Bureau, 2014 National Population Projections Tables, 2014; www.census.gov/data/tables/2014/demo/popproj/2014-summary-tables.html.

[44] McGeehin M. A., Mirabelli M., The potential impacts of climate variability and change on temperature-related morbidity and mortality in the United States. Environ. Health Perspect. 109, 185–189 (2001). 11359685 10.1289/ehp.109-1240665 PMC1240665

[45] Bobb J. F., Peng R. D., Bell M. L., Dominici F., Heat-related mortality and adaptation to heat in the United States. Environ. Health Perspect. 122, 811–816 (2014). 24780880 10.1289/ehp.1307392 PMC4123027

[46] Nordio F., Zanobetti A., Colicino E., Kloog I., Schwartz J., Changing patterns of the temperature–mortality association by time and location in the US, and implications for climate change. Environ. Int. 81, 80–86 (2015). 25965185 10.1016/j.envint.2015.04.009 PMC4780576

[47] Barreca A. I., Clay K., Deschenes O., Greenstone M., Shapiro J. S., Adapting to climate change: The remarkable decline in the US temperature-mortality relationship over the twentieth century. J. Polit. Econ. 124, 105–159 (2016).

[48] Deschênes O., Greenstone M., Climate change, mortality, and adaptation: Evidence from annual fluctuations in weather in the US. Am. Econ. J. Appl. Econ. 3, 152–185 (2011).

[49] United States Census Bureau, 2015 American Housing Survey, 2015; www.census.gov/programs-surveys/ahs/data/interactive/ahstablecreator.html).

[50] United States Energy Information Administration, 2015 Residential Energy Consumption Survey (RECS) Data, 2016; www.eia.gov/consumption/residential/data/2015/).

[51] Arbuthnott K., Hajat S., Heaviside C., Vardoulakis S., What is cold-related mortality? A multi-disciplinary perspective to inform climate change impact assessments. Environ. Int. 121, 119–129 (2018). 30199667 10.1016/j.envint.2018.08.053

[52] Tobías A., Armstrong B., Gasparrini A., Brief report: Investigating uncertainty in the minimum mortality temperature methods and application to 52 Spanish cities. Epidemiology 28, 72–76 (2017). 27748681 10.1097/EDE.0000000000000567 PMC5380105

[53] Åström D. O., Tornevi A., Ebi K. L., Rocklöv J., Forsberg B., Evolution of minimum mortality temperature in Stockholm, Sweden, 1901–2009. Environ. Health Perspect. 124, 740–744 (2016). 26566270 10.1289/ehp.1509692 PMC4892916

[54] Chung Y., Yang D., Gasparrini A., Vicedo-Cabrera A. M., Ng C. F. S., Kim Y., Honda Y., Hashizume M., Changing susceptibility to non-optimum temperatures in Japan, 1972–2012: The role of climate, demographic, and socioeconomic factors. Environ. Health Perspect. 126, 057002 (2018). 29727132 10.1289/EHP2546 PMC6071988

[55] Kinney P. L., Schwartz J., Pascal M., Petkova E., Le Tertre A., Medina S., Vautard R., Winter season mortality: Will climate warming bring benefits? Environ. Res. Lett. 10, 064016 (2015). 26495037 10.1088/1748-9326/10/6/064016 PMC4610409

[56] Ebi K. L., Mills D., Winter mortality in a warming climate: A reassessment. Wiley Interdiscip. Clim. Change 4, 203–212 (2013).

[57] Gasparrini A., Modeling exposure–lag–response associations with distributed lag non-linear models. Stat. Med. 33, 881–899 (2014). 24027094 10.1002/sim.5963 PMC4098103

[58] Rocklöv J., Ebi K. L., High dose extrapolation in climate change projections of heat-related mortality. J. Agric. Biol. Environ. Stat. 17, 461–475 (2012).

[59] Curriero F. C., Heiner K. S., Samet J. M., Zeger S. L., Strug L., Patz J. A., Temperature and mortality in 11 cities of the eastern United States. Am. J. Epidemiol. 155, 80–87 (2002). 11772788 10.1093/aje/155.1.80

[60] Massey N., Jones R., Otto F. E. L., Aina T., Wilson S., Murphy J. M., Hassell D., Yamazaki Y. H., Allen M. R., Weather@home—Development and validation of a very large ensemble modelling system for probabilistic event attribution. Q. J. Roy. Meteorol. Soc. 141, 1528–1545 (2015).

[61] Mitchell D., AchutaRao K., Allen M., Bethke I., Beyerle U., Ciavarella A., Forster P. M., Fuglestvedt J., Gillett N., Haustein K., Ingram W., Iversen T., Kharin V., Klingaman N., Massey N., Fischer E., Schleussner C.-F., Scinocca J., Seland Ø., Shiogama H., Shuckburgh E., Sparrow S., Stone D., Uhe P., Wallom D., Wehner M., Zaaboul R., Half a degree additional warming, prognosis and projected impacts (HAPPI): Background and experimental design. Geosci. Model Dev. 10, 571–583 (2017).

[62] Taylor K. E., Stouffer R. J., Meehl G. A., An overview of CMIP5 and the experiment design. Bull. Am. Meteorol. Soc. 93, 485–498 (2012).

[63] Hempel S., Frieler K., Warszawski L., Schewe J., Piontek F., A trend-preserving bias correction – the ISI-MIP approach. Earth Syst. Dynam. 4, 219–236 (2013).

