# Supplementary material for: Increasing mitigation ambition to meet the Paris Agreement’s temperature goal avoids substantial heat-related mortality in U.S. cities
Source: Sci Adv. 2019 Jun 5;5(6):eaau4373. doi: 10.1126/sciadv.aau4373 (PMC6551192; doi:10.1126/sciadv.aau4373)
Supplement: http://advances.sciencemag.org/cgi/content/full/5/6/eaau4373/DC1 [file supp_5_6_eaau4373__index.html]

Science Advances | Science Advances

## Supplementary Materials

**This PDF file includes:**

- Fig. S1. Estimated exposure-response relationships between daily mean temperature and all-cause mortality over selected U.S. cities.
- Fig. S2. One-in-30-year heat-related mortality per 100,000 persons that is avoidable by stabilizing future warming at the 1.5° and 2°C Paris Agreement thresholds rather than 3°C.
- Fig. S3. Heat-related mortality return period curves in future stabilization scenarios of 1.5°, 2°, and 3°C.
- Fig. S4. Population-normalized heat-related mortality return period curves in future stabilization scenarios of 1.5°, 2°, and 3°C.
- Table S1. The MMT and its percentile rank in the 1987–2000 observations in each city.
- Table S2. Maximum observed and projected temperatures and the percentage of days on which the projected temperature exceeds the maximum observed temperature in each scenario and city.

Download PDF

**Files in this Data Supplement:**

- Adobe PDF - aau4373\_SM.pdf
