# Supplementary material for: Increasing mitigation ambition to meet the Paris Agreement’s temperature goal avoids substantial heat-related mortality in U.S. cities
Source: Sci Adv. 2019 Jun 5;5(6):eaau4373. doi: 10.1126/sciadv.aau4373 (PMC6551192; doi:10.1126/sciadv.aau4373)
Supplement: Download PDF [file aau4373_SM.pdf]

Science Advances
AAAS

advances.sciencemag.org/cgi/content/full/5/6/eaau4373/DC1

Supplementary Materials for

Increasing mitigation ambition to meet the Paris Agreement's temperature goal avoids substantial heat-related mortality in U.S. cities

Y. T. Eunice Lo*, Daniel M. Mitchell, Antonio Gasparrini, Ana M. Vicedo-Cabrera, Kristie L. Ebi, Peter C. Frumhoff, Richard J. Millar, William Roberts, Francesco Sera, Sarah Sparrow, Peter Uhe, Gethin Williams

*Corresponding author. Email: eunice.lo@bristol.ac.uk

Published 5 June 2019, *Sci. Adv.* **5**, eaau4373 (2019)
DOI: 10.1126/sciadv.aau4373

This PDF file includes:

Fig. S1. Estimated exposure-response relationships between daily mean temperature and all-cause mortality over selected U.S. cities.
Fig. S2. One-in-30-year heat-related mortality per 100,000 persons that is avoidable by stabilizing future warming at the 1.5° and 2°C Paris Agreement thresholds rather than 3°C.
Fig. S3. Heat-related mortality return period curves in future stabilization scenarios of 1.5°, 2°, and 3°C.
Fig. S4. Population-normalized heat-related mortality return period curves in future stabilization scenarios of 1.5°, 2°, and 3°C.
Table S1. The MMT and its percentile rank in the 1987–2000 observations in each city.
Table S2. Maximum observed and projected temperatures and the percentage of days on which the projected temperature exceeds the maximum observed temperature in each scenario and city.

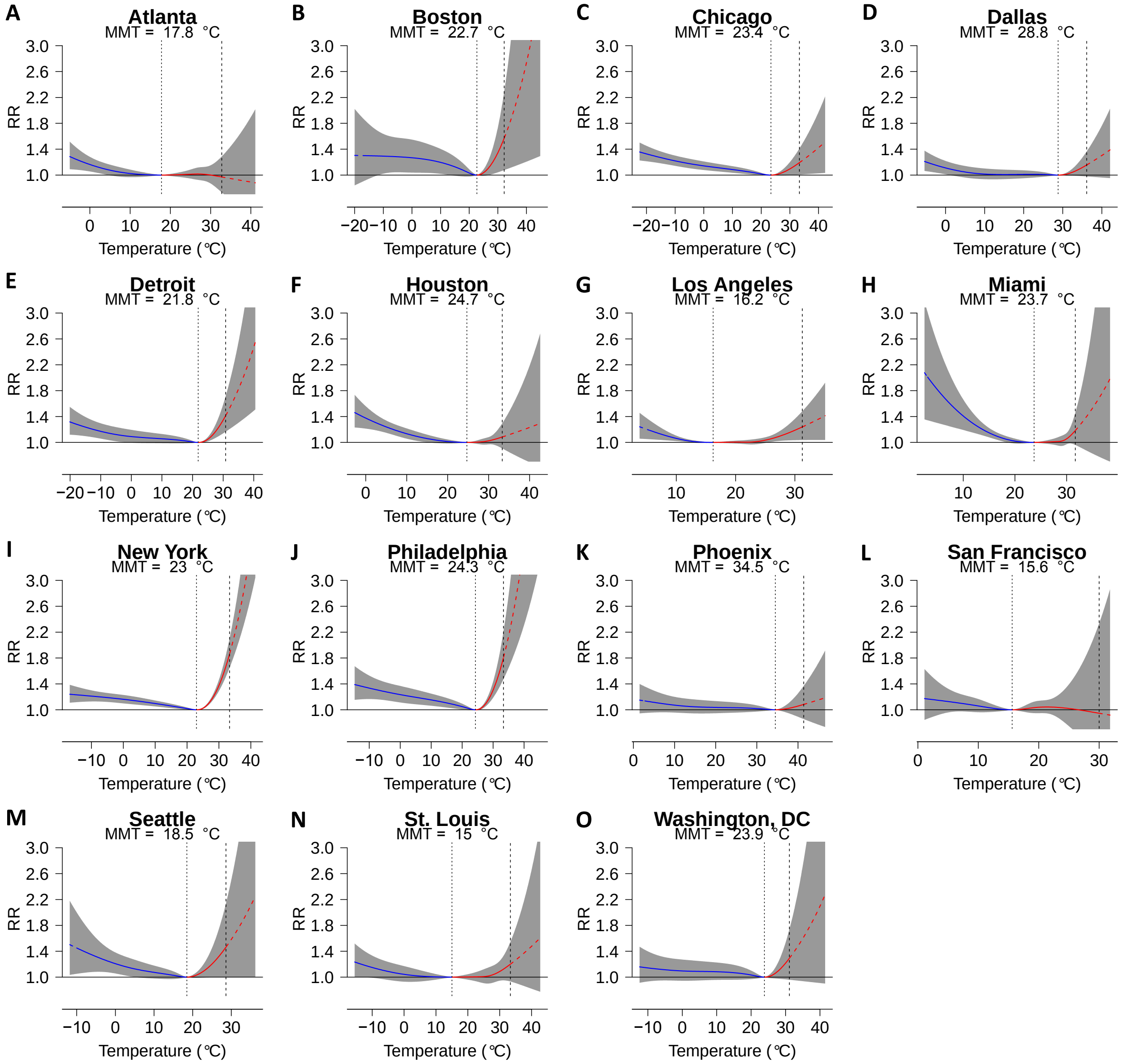

Fig. S1. Estimated exposure-response relationships between daily mean temperature and all-cause mortality over selected U.S. cities. The blue and red lines show the relative mortality risk associated with low and high temperatures, respectively. The grey shading shows the 95% empirical confidence interval of the corresponding exposure-response relationship. The dotted vertical line on each graph indicates the location-specific minimum mortality temperature (MMT), whereas the dashed vertical line indicates the highest observed daily mean temperature during 1987–2000 over each city.

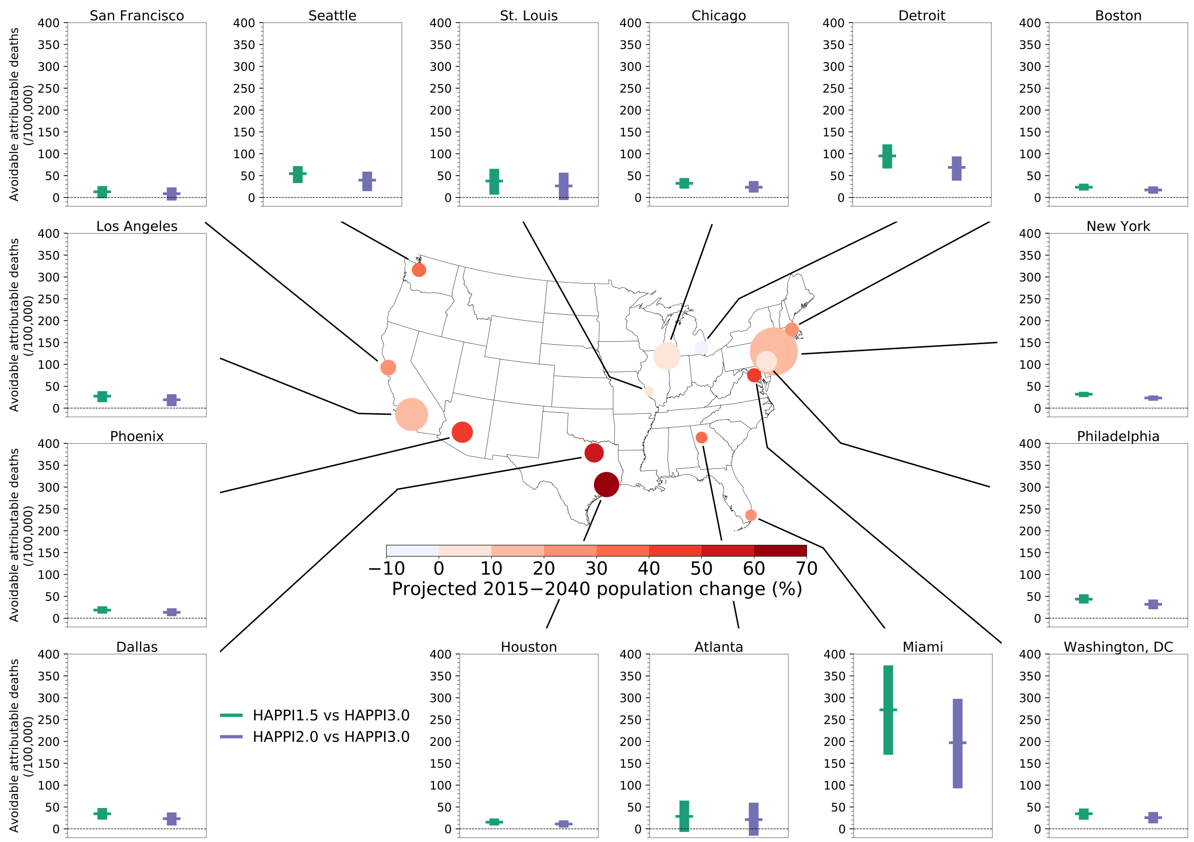

Fig. S2. One-in-30-year heat-related mortality per 100,000 persons that is avoidable by stabilizing future warming at the 1.5° and 2°C Paris Agreement thresholds rather than 3°C. The point estimates show the mean 1-in-30-year mortality level across 100 plausible exposure-response relationships, whereas the error bars show the 95% empirical confidence interval accounting for uncertainties from internal climate variability and the exposure-response relationship. All estimates are normalized by the cities' July 2016 population, the square root of which is proportional to the size of the bubbles on the central map. Confidence intervals that do not include 0 (dotted line on each panel) indicate a statistically significant number of avoidable deaths. The color of each bubble indicates the city's projected population change between 2015 and 2040.

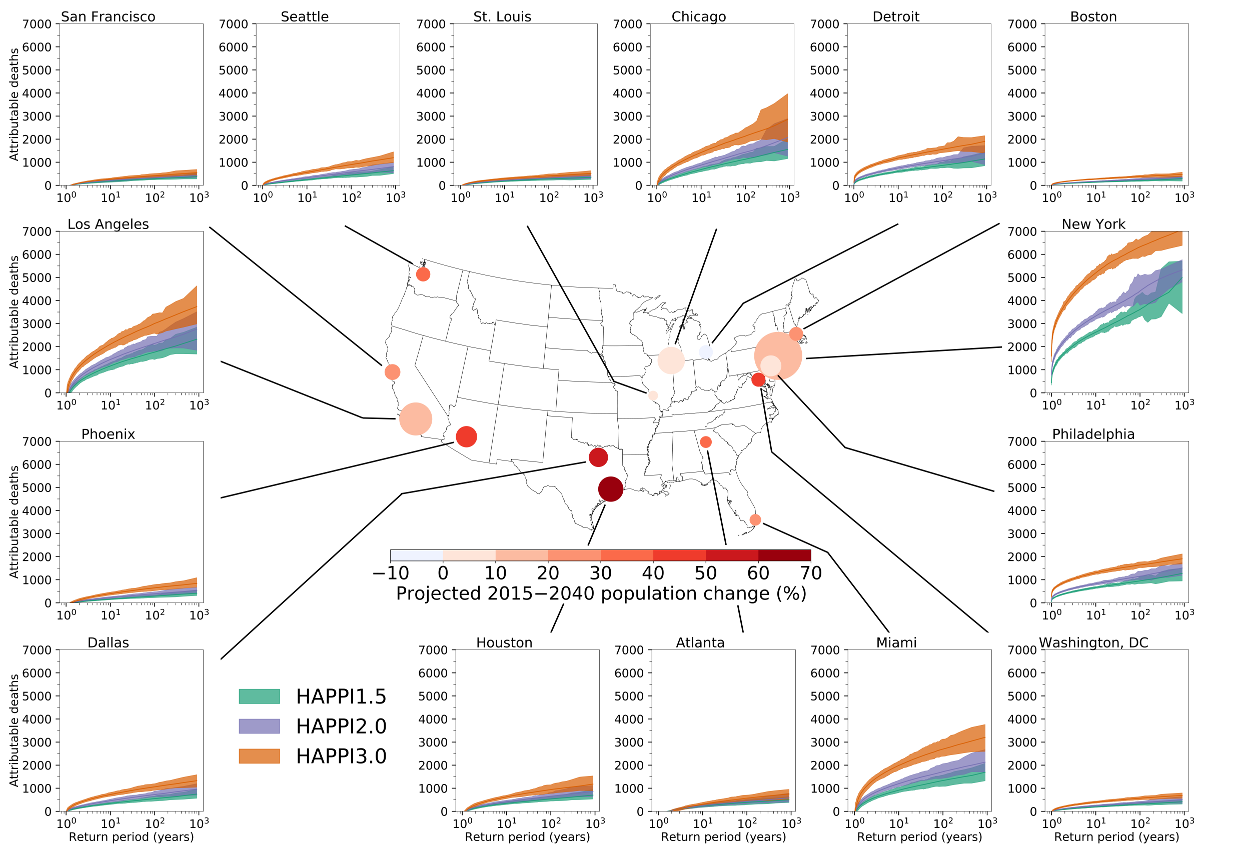

Fig. S3. Heat-related mortality return period curves in future stabilization scenarios of 1.5°, 2°, and 3°C. Mortality is expressed as the cumulated number of annual deaths attributable to high temperatures over the 900-year simulation for each scenario from the 90-member ensemble, under the assumption of constant population. The solid lines are the mean return period curve across 101 plausible exposure-response relationships. The shadings show the 95% empirical confidence intervals that include the uncertainty on the exposure-response relationship and the uncertainty arising from internal climate variability (computed from bootstrapping). The size of each bubble on the central map is proportional to the square root of the city's population in July 2016. The colors of the bubbles indicate the cities' projected population change between 2015 and 2040.

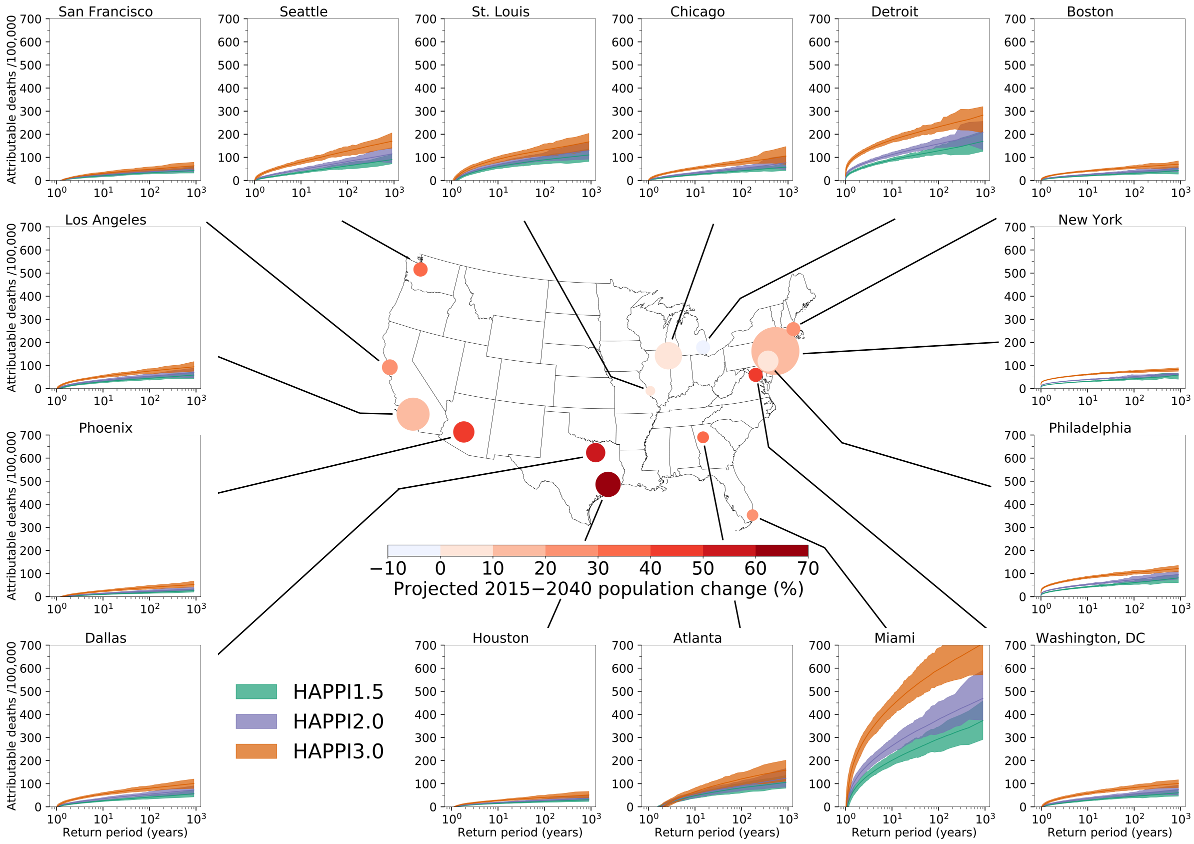

Fig. S4. Population-normalized heat-related mortality return period curves in future stabilization scenarios of 1.5°, 2°, and 3°C. Mortality is expressed as the cumulated number of annual deaths per 100,000 persons that are attributable to high temperatures over the 900-year simulation for each scenario from the 90-member ensemble. The solid lines are the mean return period curve across 101 plausible exposure-response relationships. The shadings show the 95% empirical confidence intervals that include the uncertainty on the exposure-response relationship and the uncertainty arising from internal climate variability (computed from bootstrapping). The size of each bubble on the central map is proportional to the square root of the city's population in July 2016. The colors of the bubbles indicate the cities' projected population change between 2015 and 2040.

Table S1. The MMT and its percentile rank in the 1987–2000 observations in each city.

City	MMT (°C)	Percentile
Atlanta	17.8	50
Boston	22.7	88
Chicago	23.4	89
Dallas	28.8	83
Detroit	21.8	84
Houston	24.7	61
Los Angeles	16.2	35
Miami	23.7	30
New York	23.0	83
Philadelphia	24.3	84
Phoenix	34.5	89
San Francisco	15.6	61
Seattle	18.5	88
St. Louis	15.0	51
Washington, DC	23.9	84

Table S2. Maximum observed and projected temperatures and the percentage of days on which the projected temperature exceeds the maximum observed temperature in each scenario and city. Obs T_{max} represents the maximum observed temperature during the 1987–2000 period. HAPPI T_{max} represents the maximum projected temperature in each bias-corrected HAPPI experiment (10 years of simulation × 90 ensemble members). % days represents the percentage of days in the 900 model years in each HAPPI experiment on which the daily mean temperature exceeds the maximum observed temperature.

City	Obs	HAPPI1.5		HAPPI2.0		HAPPI3.0	
	T_{max} (°C)	T_{max} (°C)	% days	T_{max} (°C)	% days	T_{max} (°C)	% days
Atlanta	32.8	39.5	1.4	39.7	2.4	41.1	6.5
Boston	32.2	41.2	0.6	42.8	0.9	44.8	2.2
Chicago	33.3	39.4	0.5	40.7	1.1	42.3	3.2
Dallas	36.1	40.4	0.3	40.6	0.7	42.1	2.4
Detroit	30.8	37.3	1.3	38.0	2.2	40.5	5.6
Houston	33.3	40.3	1.1	42.1	1.8	42.6	4.4
Los Angeles	31.3	33.9	0.0	33.5	0.0	35.1	0.3
Miami	31.7	37.2	3.4	38.2	7.7	38.4	21.7
New York	33.3	39.3	0.4	40.3	0.7	41.4	2.2
Philadelphia	33.3	41.4	0.9	42.0	1.5	45.2	3.8
Phoenix	41.4	44.2	0.1	44.8	0.4	46.6	2.0
San Francisco	30.0	30.3	0.0	30.6	0.0	31.8	0.0
Seattle	28.6	33.6	0.1	33.4	0.3	36.2	1.4
St. Louis	33.3	40.9	2.4	41.2	4.1	42.7	8.5
Washington, DC	31.1	39.2	1.9	41.4	3.1	41.5	7.0
